# INFLAMMATORY CYTOKINE, SOCIAL SUPPORT, AND HEALTH DISPARITIES IN PERSONS WITH COGNITIVE IMPAIRMENT AND DEMENTIA

**DOI:** 10.1093/geroni/igad104.2752

**Published:** 2023-12-21

**Authors:** Ayse Malatyali, Tom Cidav, Rui Xie, Ladda Thiamwong

**Affiliations:** University of Central Florida, Orlando, Florida, United States; The Johns Hopkins University School of Medicine, Baltimore, Maryland, United States; University of Central Florida, Orlando, Florida, United States; University of Central Florida, Orlando, Florida, United States

## Abstract

The protective role of social support on cognitive functioning is extensively documented. A pro-inflammatory cytokine, Tumor necrosis factor-alpha (Tnf-alpha), might have a role in the connection between social support and dementia as a marker of systemic inflammation. This study describes the influence of lack of friends’ support and Tnf-alpha on cognitive functioning. We conducted a cross-sectional study on Health and Retirement Study participants aged 65 and above, using datasets from the 2016 interviews. Of the 9,262 participants, 72.4% had normal cognition. 21.3% had cognitive impairment, and 6.3% had dementia. Our regression model controlled with depression scores revealed that participants with a lack of friends’ support were 51% more likely to have cognitive impairment and 54% more likely to have dementia. The effect of Tnf-alpha was comparably higher on participants with dementia and cognitive impairment than on participants with normal cognition. Compared to quartile-1 (lowest Tnf-alpha levels), Quartile-4 (highest Tnf-alpha levels) was significantly associated with cognitive impairment (OR=1.60) and dementia (OR=2.37). Quartile-3 was only a significant predictor of dementia (OR=2.08). The effect of Tnf-alpha was insignificant in quartile-2 for both groups. Hispanics were 65% more, and African Americans were 67% more likely to have cognitive impairment than non-Hispanic whites. The likelihood of having dementia was even higher in racial/ethnic minorities: Hispanics were 3.3 times more, and African Americans were 2.3 times more likely to have dementia than non-Hispanic whites. Future research can focus on racial and ethnic variations in pro-inflammatory cytokines and social support among people with dementia.

